# Genetic diversity and genetic relatedness in *Plasmodium falciparum* parasite population in individuals with uncomplicated malaria based on microsatellite typing in Eastern and Western regions of Uganda, 2019–2020

**DOI:** 10.1186/s12936-021-03763-6

**Published:** 2021-05-31

**Authors:** Agaba B. Bosco, Karen Anderson, Karryn Gresty, Christiane Prosser, David Smith, Joaniter I. Nankabirwa, Sam Nsobya, Adoke Yeka, Rhoda Namubiru, Emmanuel Arinaitwe, Paul Mbaka, John Kissa, Chae Seung Lim, Charles Karamagi, Joan K. Nakayaga, Moses R. Kamya, Qin Cheng

**Affiliations:** 1 College of Health Sciences, Makerere University, Kampala, Uganda; 2 National Malaria Control Division, Kampala, Uganda; 3 QIMR Berghofer Medical Research Institute, Brisbane, QLD Australia; 4 Australian Defence Force Malaria and Infectious Disease Institute, Brisbane, Australia; 5 Infectious Diseases Research Collaboration, Kampala, Uganda; 6 World Health Organization Country Office, Kampala, Uganda; 7 National Health Information Division, Ministry of Health, Kampala, Uganda; 8 Department of Laboratory Medicine, College of Health Sciences, Korea University, Seoul, South Korea

**Keywords:** Malaria, Rapid diagnostic tests, Genetic diversity, Multiplicity of infection, Multiclonal infections, Parasite relatedness, *Pfhrp2* deletion, Microsatellite markers

## Abstract

**Background:**

Genetic diversity and parasite relatedness are essential parameters for assessing impact of interventions and understanding transmission dynamics of malaria parasites, however data on its status in *Plasmodium falciparum* populations in Uganda is limited. Microsatellite markers and DNA sequencing were used to determine diversity and molecular characterization of *P. falciparum* parasite populations in Uganda.

**Methods:**

A total of 147 *P. falciparum* genomic DNA samples collected from cross-sectional surveys in symptomatic individuals of 2–10 years were characterized by genotyping of seven highly polymorphic neutral microsatellite markers (n = 85) and genetic sequencing of the Histidine Rich Protein 2 (*pfhrp2*) gene (n = 62). ArcGIS was used to map the geographical distribution of isolates while statistical testing was done using Student's t-test or Wilcoxon's rank-sum test and Fisher’s exact test as appropriate at P ≤ 0.05.

**Results:**

Overall, 75.5% (95% CI 61.1–85.8) and 24.5% (95% CI14.2–38.9) of parasites examined were of multiclonal (mixed genotype) and single clone infections, respectively. Multiclonal infections occurred more frequently in the Eastern region 73.7% (95% CI 48.8–89.1), P < 0.05. Overall, multiplicity of infection (MOI) was 1.9 (95% CI 1.7–2.1), P = 0.01 that was similar between age groups (1.8 vs 1.9), P = 0.60 and regions (1.9 vs 1.8), P = 0.43 for the < 5 and ≥ 5 years and Eastern and Western regions, respectively. Genomic sequencing of the *pfhrp2* exon2 revealed a high level of genetic diversity reflected in 96.8% (60/62) unique sequence types. Repeat type AHHAAAHHATD and HRP2 sequence Type C were more frequent in RDT−/PCR + samples (1.9% vs 1.5%) and (13% vs 8%), P < 0.05 respectively. Genetic relatedness analysis revealed small clusters of gene deleted parasites in Uganda, but no clustering with Eritrean parasites.

**Conclusion:**

High level of genetic diversity of *P. falciparum* parasites reflected in the frequency of multiclonal infections, multiplicity of infection and variability of the *pfhrp2* gene observed in this study is consistent with the high malaria transmission intensity in these settings. Parasite genetic analysis suggested spontaneous emergence and clonal expansion of *pfhrp2* deleted parasites that require close monitoring to inform national malaria diagnosis and case management policies.

**Supplementary Information:**

The online version contains supplementary material available at 10.1186/s12936-021-03763-6.

## Background

In 2019, the World Health Organization (WHO) estimated that there were 229 million cases of and 409,000 deaths due to malaria globally. The WHO African region accounts for a disproportionately high share of the global burden (94% of malaria cases in 2019 alone) [1, 2]. Nearly all malaria cases in the WHO African region are caused by *Plasmodium falciparum.* Uganda is ranked among the top six countries with the highest malaria burden [1–3]. Despite the improved coverage of interventions, malaria remains a major public health problem in Uganda accounting for 30% of outpatient visits to health facilities (HF), 14–20% of hospital admissions and 8–10% of inpatient deaths [4–6]. Although the epidemiology of malaria is heterogenous and varies in space and time, it is endemic throughout the whole country, and transmission occurs year-round. *Plasmodium falciparum* accounts for > 95% of malaria infections in Uganda [6–11]. In the past 10 years, the Uganda national malaria programme registered great milestones in reducing malaria transmission as shown by the last three national population-based malaria indictor surveys [5, 11, 12]. There is evidence of declining parasite prevalence from 44% in 2009 to 19% in 2014 and 9.1% in 2019 [5, 11, 12]. Based on this evidence, the country developed an ambitious malaria control and elimination strategic plan 2021–2025 that aims to further reduce parasite prevalence to less than 2% by 2025 including transformation of targeted districts from control to elimination phase [13, 14]. This requires enhanced monitoring the impact of the malaria control interventions.

Individuals living in high malaria transmission settings often carry several genetically distinct clones of the same parasite species (multiclonal infection) that can be the result of independent bites of infected mosquitoes (also called superinfection), or a single mosquito bite transmitting a genetically diverse sporozoite inoculum. As seen elsewhere, the number of co-infections (multiclonal) within a host might be an important indicator of transmission intensity and annual incidence rates (API) and, therefore, a measure of impact of interventions [15] and may have negative effect on treatment and drug efficacy trials, diagnosis and surveillance.

The high degree of genetic diversity within the *P. falciparum* HRP2 protein antigen epitopes can potentially affect the functionality of HRP-based rapid diagnostic tests (RDTs) that currently account for > 85% total malaria testing in Uganda [16–20]. A number of studies to date have documented extensive length, size and amino acid structural variations in the exon 2 of *pfhrp2* gene between parasite strains [16, 20]. The *pfhrp2* exon2 protein is composed of varying numbers of different types of amino acid repeat motifs (sequences) with each motif having variable copies, which give rise to large variation in the size of PCR fragments that may pose parasite detection challenges with the current HRP2 RDTs [16–18, 20]. Therefore, understanding genetic diversity, genetic relatedness between parasite population structures and transmission dynamics in *P. falciparum* populations could provide important information to inform current and future malaria control intervention efforts in Uganda.

Accurate measurement and estimation of genetic diversity and parasite relatedness require identification of all clones in multiclonal infections. However identification of the within-host parasite component in multiclonal infection is often difficult due to lack of appropriate molecular diagnostic tools [15]. Previous investigations of genetic diversity and parasite relatedness in Uganda have mostly relied on size-polymorphic antigenic markers such as MSP1, MSP2 and GLURP that can be amplified by PCR and the size of the amplicon determined by either gel or capillary electrophoresis. However, these molecular markers are under strong immune selection which affects the distribution of alleles in the population. Selection pressure could bias diversity measurements with these markers, especially in endemic areas where individuals could harbour several parasite clones [15]. Also the limited discrimination of alleles of similar sizes on agarose gel electrophoresis makes it difficult to discriminate allele size difference of less than 20 bp resulting in underestimation of diversity and multiplicity based on polymorphic genes [15, 16, 20]. Amplicon ultra-deep sequencing and whole genome sequencing techniques have high sensitivity and specificity particularly when detecting minority clones in cases of multiclonal infections. However, the costs and time factors attached to the use of these techniques are relatively high thereby limiting their application especially in resource limited settings.

Microsatellite markers are highly polymorphic with tandem repeats of 2–6 bp, abundant and widely distributed throughout the *Plasmodium* parasite genome [15, 16, 21]. Microsatellites are expected to be selection neutral with no phenotypic consequences, tend to be more reliably variable in multiple populations than other markers and are readily amplifiable using relatively cheaper methods, such as PCR [15–17, 20–22]. In this study, highly polymorphic microsatellite markers were deployed to investigate the genetic diversity, multiplicity of infections and parasite relatedness in a set of *P. falciparum* isolates collected from the different geographical settings in Uganda that have been confirmed with presence of mutant parasites carrying *pfhrp2/3* gene deletions.

## Methods

### Study design and setting

*Plasmodium falciparum* genomic DNA samples that were available from a previous study that characterized the status of *pfhrp2* and *pfhrp3* parasite genes were analysed [23]. Details of the study population, data collection and DBS sample collection have been published elsewhere [24–26]. Details of genomic DNA extraction method was also published elsewhere [23].

### Origin and selection of genomic DNA samples

**Fig. 1:**
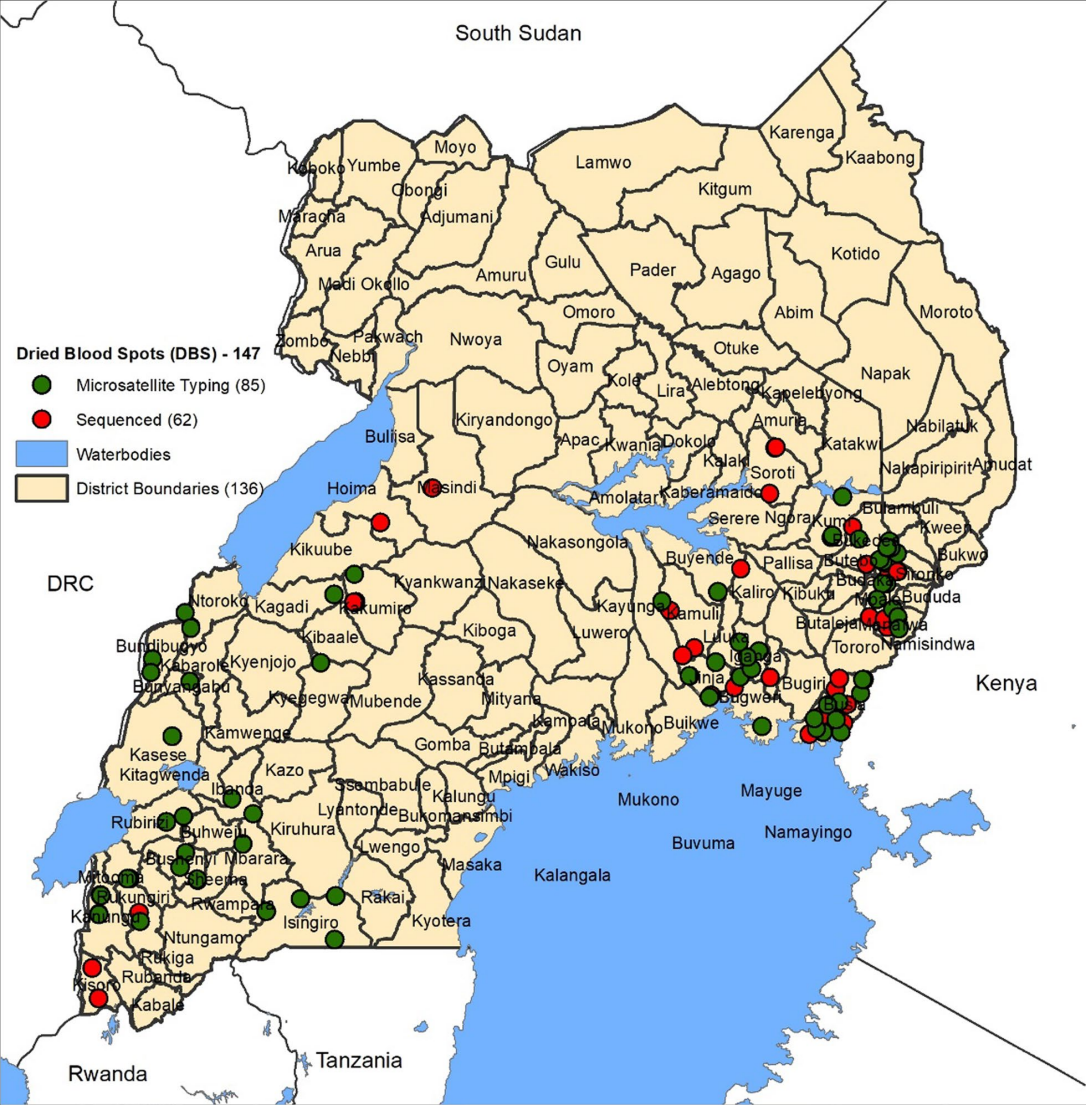
Geographical distribution of DNA samples used for genotyping. The red and green dots indicate the location where the sequenced and microsatellite typed samples were collected respectively. Black lines indicate district boundaries

The available DBS samples consisted of 160 (microscopy + /RDT−) and 140 (microscopy + /RDT +) samples whose genomic DNA had been extracted for *pfhrp2/3* gene deletion study [23]. From these DNA samples, 85 were randomly selected from different *pfhrp2/3* status groups and used for microsatellite genotyping. An additional 62 samples were randomly selected from the *pfhrp2* positive sample group to undergo Sanger sequencing. The geographical distribution of the 85 microsatellite and 62 sequencing samples is shown in Fig. 1.

### Laboratory analysis

#### Microsatellites genotyping of *P. falciparum*

Genotyping of the same 7 neutral microsatellite loci/markers previously analysed to reveal genetic relatedness of parasites with and without gene deletions in South America [27] and Africa [28] was conducted for each of the 85 samples as previously described [27]. Locations of these markers have been described previously [27, 29, 30]. The microsatellite markers TA1, PolyA, PfPK2, TA109 and 2490 were semi-nested PCRs while 313 and 383 were single round PCR reactions. Briefly, the primary reaction for each marker was carried out in a solution of total volume 15 µL containing 4.7 µL Nuclease Free Water, 1.8 µL MgCl_2_ (25 mM), 1.5 µL 10 × Reaction Buffer II, 0.6 µL dNTPs (1.25 mM), 0.6 µL Forward Primer(10 μM), 0.6 µL Reverse Primer(10 μM), 0.25 µL AmpliTaq Gold (5U/μL), and 5 µL DNA Template. Round 1 PCR conditions were 94 °C for 5 min, (94 °C for 30 s, 42 °C for 30 s, 40 °C for 65 s) 25 cycles, 65 °C for 5 min. The secondary reaction contained the same reagents as the primary reaction for each marker with the exception of the 0.6 µL each of the labelled primer pair. TA1-F, TA109-F, 2490-R and 313-F primers were labelled with -6-FAM. PolyA-R primer was labelled with –PET, PK2-R primer with -NED and 383-F primer with –VIC. One µL of the primary reaction product was used in a final volume of 15 µL in the semi-nested PCR reactions. Round 2 PCR conditions were 94 °C for 5 min, (94 °C for 30 s, 45 °C for 30 s, 40 °C for 65 s) 25 cycles, 65 °C for 5 min.

PCR conditions for the 313 and 383 PCRs were as follows: 94 °C for 5 min, (94 °C for 30 s, 50 °C for 30 s, 60 °C for 30 s) for 5 cycles, (94 °C for 30 s, 45 °C for 30 s, 60 °C for 30 s) for 40 cycles, then 60 °C for 2 min. A 5µL sample of the PCR product was then run on a 2% agarose gel to determine the factor prior to assay on the sequencer. Fluorescently labelled PCR products were assayed for size on an ABI 3100 Genetic Analyzer sequencer (Applied Biosystems, Foster City, CA, USA). The fragments/alleles were scored manually using Peak Scanner Software version 1.0 (Applied Biosystems), using a height of 300 relative fluorescence units as the minimal peak threshold (bands smaller than 300 relative fluorescence units (rfu) were defined as background). For samples producing more than one peak, peaks with height of > 300 rfu and also > 30% of the highest peak are classified as positive peaks [28]. Samples for which no amplification was obtained in some markers were repeated and re-analysed or excluded if they failed to amplify more than 3 of the 7 markers the second time [27, 28]. A laboratory *P. falciparum* line of 3D7 was included in each run as a control and sizes of seven microsatellite markers were calibrated against those of 3D7 before conducting genetic relatedness analysis.

#### Polyclonality

Samples were classified as either single clone infections (containing only one allele per marker at all 7 markers) or multiclonal/polyclonal infections (containing two or more alleles at any one or more of the 7 markers. The proportion of polyclonal infections were calculated as: number of samples with polyclonal infections/number of samples typed × 100%.

#### Multiplicity of infection (MOI)

MOI for each of the 7 microsatellite markers was calculated as: the total number of alleles revealed /total number of samples typed for that marker. The overall MOI was calculated as: the sum of the highest number of alleles at any of the 7 microsatellite markers typed for all samples/the total number of samples typed.

#### Haplotype construction

Seven-microsatellite marker haplotypes were constructed for samples with single clone infections or multiple clone infections with multiple alleles only detected on one of the 7 markers. For a proportion of samples where microsatellite data were obtained only for five or six of the seven markers typed, five- or six-microsatellite markers haplotypes were constructed.

#### Genetic relatedness of parasites

Five to seven microsatellite haplotypes were used to determine the relatedness among Ugandan parasite isolates and between parasites from Uganda and Eritrea by using the PHYLOViZ software (www.phyloviz.net, version 1.1) [28]. Sample data including individual microsatellite size, *pfhrp2/3* status and geographical origin of the samples were imported into the online software for analysis. Plots were produced using Phyloviz software at cutoff value of 3 minimum differences for 4 loci).

#### *Pfhrp2* DNA and putative amino acid sequence analyses

Exon 2 of the *pfhrp2* gene was amplified and sequenced as previously described Baker et al*.* [20]. DNA sequences from samples with single clone infections or from multiple clone infections but producing clear sequences for a dominant clone (one that masks or overrides other variants) were used for sequence diversity analysis. Putative amino acid repeat types and HRP2 sequence types were determined following Baker et al*.* [16]. DNA sequences were imported into MEGA4.0.2 software [28] and were used to generate phylogeny bootstrap consensus neighbor-join tree using the software. Exon1 of the *pfhrp2* gene was not sequenced as exon1 encodes a conserved signal peptide.

### Data management and statistical analysis

Demographics and variables linked to the genomic DNA samples were extracted from the previous survey database. All data were entered and managed in Excel before it was exported to STATA for analysis. ArcGIS software version 10.8, Environmental Systems Research Institute (Esri), California U.S) was used to map the geographical distribution, locations and sites where the genomic DNA samples were collected and where the different *pfhrp2* amino acid sequences/types actually occurred. Data analysis was done with STATA Ver 14, college Station, Tx: StataCorp LP), GraphPad Prism 7.00 for Windows (GraphPad Software, La Jolla, CA, USA). Descriptive analysis was done for baseline characteristics and presented as proportions. Proportions of the different sequence types were assessed for statistical significance using the Student's t-test or Wilcoxon's rank-sum test as appropriate while MOIs and multi-clones were analysed with Fisher’s exact test at level of significance p ≤ 0.05.

### Ethical approval

The study was approved by the Makerere University School of Medicine Research and Ethics committee (#REC REF 2017-111), the Uganda National Council of science and technology (Ref No: HS271ES), and the Australian Department of Defence and Veterans’ Affairs Human Research Ethics Committee (DDVA HREC 096–18). In the primary surveys from where samples were collected, participants were enrolled after providing written informed consent for future use of biological samples for molecular analysis.

## Results

### Characterization and profile of samples

A total of 147 PCR confirmed parasite genomic DNA samples were available for molecular characterization in this study. Seven-microsatellite-marker typing was performed for 85 samples, however only 49 samples produced positive allele peaks at > 5 microsatellite markers and were considered for analysis. Thirty-six samples (n = 36) that produced allele peaks at 1–4 microsatellite markers only were excluded from further analyses on MOIs and parasite genetic relatedness. Clean DNA sequences of the *pfhrp2* exon 2 was obtained from 62 samples. The entire sample of 147 genomic DNA and their demographics came from a study that previously characterized the *pfhpr2* and *pfhrp3* genes [23].

### Baseline characteristics samples

The majority of these samples was from males (54.4%), aged ≥ 5 years (65.9%) and from the eastern region of Uganda (53.7%). Most had a parasite density ≥ 1000/μl (65.3%). All the genomic DNA were from PCR confirmed *P. falciparum* infected samples (Table 1).

**Table 1:** Baseline characteristics of samples

Variable	Frequency	Proportion (%)	Sequenced (%)	Microsatellite typing (%)
	Total (n = 147)		(n = 62)	(n = 85)
Age				
< 5	50	34.01	40.3	29.4
≥ 5	97	65.99	59.6	70.6
Gender				
Male	80	54.42	54.8	54.1
Female	67	45.58	45.2	35.9
Region				
Western	68	46.3	37.1	51.7
Eastern	79	53.7	62.9	48.2
Density				
< 1000	51	34.69	38.7	45.8
≥ 1000	96	65.31	61.3	54.1
Endemicity				
Low transmission	87	59.18	45.2	69.4
Moderate	60	40.82	54.8	30.9

< 5 and ≥ 5 means less than 5 and equal or above years of age respectively. < 1,000 and ≥ 1,000 means parasite densities of less than 1,000 and equal or greater than 1,000 parasites/µL, respectively

### Polyclonality of infections

Microsatellite typing revealed a high proportion samples examined had more than one allele on at least one of the seven markers indicative of multi-clone infections. Overall, 75.51 (95%, CI61.1–85.8) of samples examined were classified as multiple-clone (mixed genotype) infections, significantly higher compared to single clone 24.5% (95% CI 14.2–38.9), P < 0.05. This high level of polyclonality in samples indicates frequent infections consistent with high malaria transmission intensity in the study areas. Although not statistically significant, proportions of multiclonal infections were 71.8% (91% CI 53.4–85.1) and 82.4% (95% CI 55.5–94.6), P > 0.05 in > 5 and ≥ 5 years respectively. Multiclonal infections significantly occurred in isolates collected from Eastern 73.7% (95% CI 48.8–89.1) compared to Western region 26.3% (95% CI 10.9–51.2), P < 0.05.

### Multiplicity of infection

The number of alleles at any of the 7 markers varied between markers. The highest number of alleles at any marker was 4 (Table 2). MOI at individual marker varied between 1 and 1.51. The overall MOI in this set of samples was 1.92 (95% CI1.72–2.12), consistent with high transmission intensity. Though not statistically significant the MOI was higher in children < 5 than ≥ 5 years old (1.9 and 1.8), respectively. No geographical differences in MOIs were seen in parasites collected from Eastern and Western Uganda (1.9 vs 1.8), P > 0.05, respectively (Table 2). Importantly, 5/7 samples with gene deletions were multi-clone infections (*pfhrp2-/pfhrp3* +  = 2, *pfhrp2-/pfhrp3-* n = 3, *pfhrp2* + */pfhrp3*- n = 1).

**Table 2:** Microsatellite markers, Typing and overall MOI

Microsatellite marker	No. of samples typed	No. of alleles revealed	Range in no. of alleles	MOI	95%CI
2409	49	65	1–3	1.33	NA
TA1	48	56	1–2	1.17	NA
383	30	38	1–2	1.27	NA
TA109	47	69	1–4	1.47	NA
313	19	19	1–1	1	NA
PolyA	49	74	1–3	1.51	NA
PK2	42	50	1–4	1.19	NA
Overall MOI*	49	94	1–4	1.92	1.72–2.12
Overall MOI* (RDT+/PCR +)	33	65	1–3	1.97	NA
Overall MOI* (RDT−/PCR +)	16	29	1–4	1.81	NA
Overall MOI* Age					
< 5 years	26	48	1–4	1.88	1.52–2.24
≥ 5 years	23	27	1–2	1.94	1.68–2.20
Overall MOI* Region					
Western	19	36	1–2	1.89	1.54–2.25
Eastern	31	51	1–4	1.93	1.68–2.19

*Calculated by selecting the highest number of alleles at any of the markers typed. NA = not applicable

### Number of 5 to 7-microsatellite marker haplotypes

Five to 7 marker haplotypes were obtained from 29 samples: 12 single clone infected samples and 17 multi-clone infected samples where multiple alleles were observed in only one marker. This resulted in a total of 42 unique haplotypes from 29 samples. The remaining samples had multiple alleles at more than one marker resulting in haplotypes unassigned due to the complexity in number of possible haplotype combinations. The high level of diversity and multiplicity in haplotypes detected in this set of samples indicates large parasite population size consistent with high transmission intensity in the areas.

Only two of the 42 haplotypes (H29 and H31) were shared between 2 (D196 and D204) and between 3 (D130, D176 and D204) different samples. Interestingly, both these haplotypes H29 and H31 were identified from parasite samples that had both *pfhrp2* and *pfhrp3* deletions. Haplotypes H25, H37 and H38 were identified in samples determined to have deleted *pfhrp2* only, while H26 and H27 were identified in a sample determined to have deleted *pfhrp3* only (Table 3). This observation suggests that parasites having *pfhrp2* or *pfhrp3* gene deletions had a lower genetic diversity compared to parasites without gene deletions indicating clonal expansion of gene deleted parasites. Furthermore, parasites with 5 different haplotypes (H29, H31, H25, H37 and H38) and two different haplotypes (H26 and H27) were observed to have deleted the *pfhrp2* gene and *pfhrp3* gene respectively, suggesting that *pfhrp2* and *pfhrp3* gene deletions have occurred in parasites with different genetic backgrounds.

**Table 3:** Haplotype determination in pfhrp2/3 deleted samples

Haplotype	Identified in sample	*pfhrp2/3* status
H29	D196, D204	*pfhrp2−/pfhrp3−*
H31	D130, D176, D196	*pfhrp2−/pfhrp3−*
H25	D154	*pfhrp2−/pfhrp3* +
H37	D169	*pfhrp2−/pfhrp3* +
H38	D169	*pfhrp2−/pfhrp3* +
H26	D263	*pfhrp2* + */pfhrp3−*
H27	D263	*pfhrp2* + */pfhrp3−*

pfhrp2/3 detected: + ; undetected:−

### Genetic relatedness of parasites

**Fig. 2:**
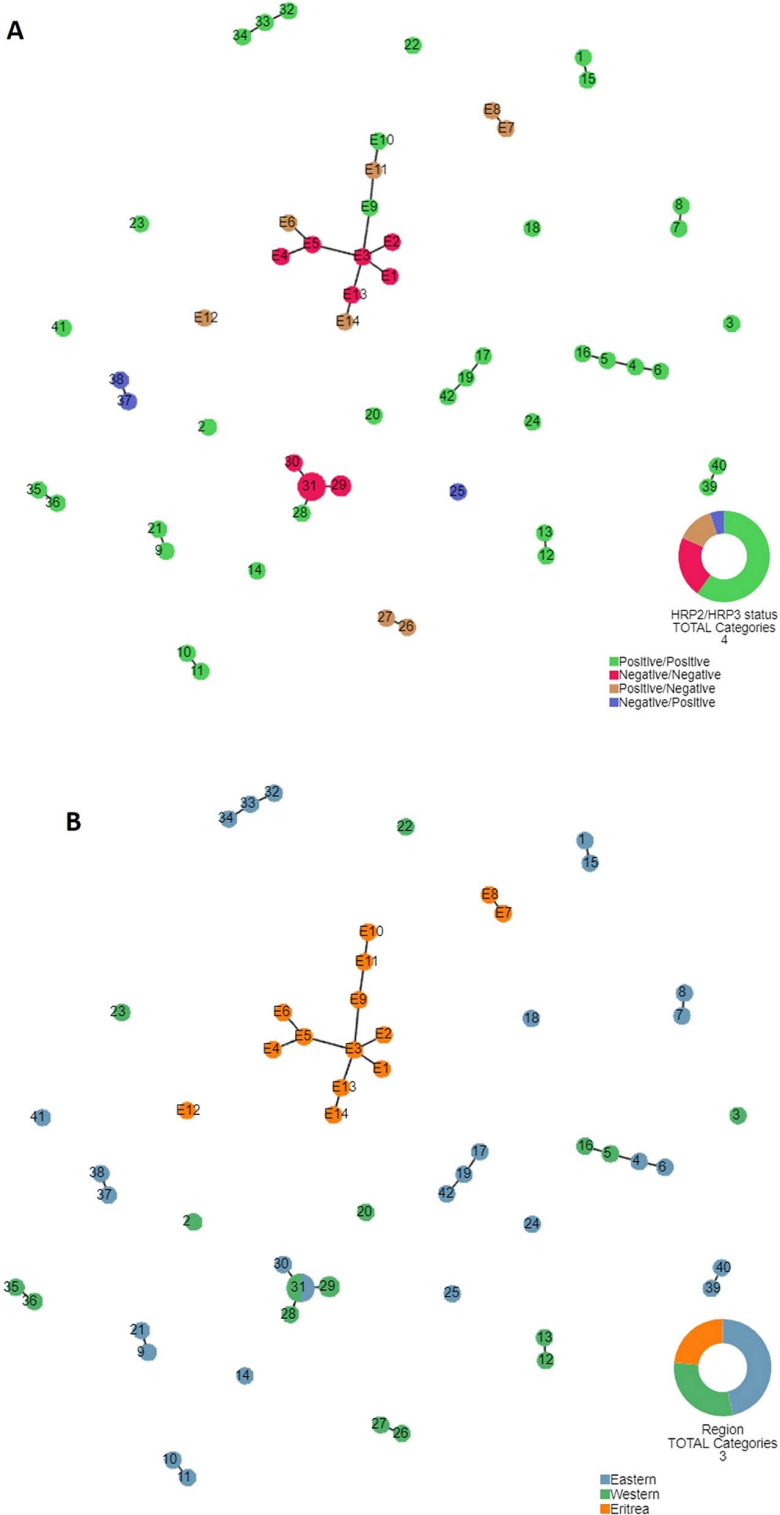
Genetic relatedness of *P. falciparum* populations. a and b Legend: Genetic relatedness of *P. falciparum* populations from Uganda differing in *pfhrp*2/3 genetic gene status (**a**) and comparison of parasite populations from Uganda and Eritrea (**b**). The relatedness among parasites was constructed using seven neutral microsatellite markers/loci indicated in Additional file 1: Table S1. Plots were produced using Phyloviz software at cutoff value of 3 minimum differences for 4 loci). Numbered circles indicate specific haplotypes. Circle sizes are proportional to the number of samples with a particular haplotype. The number of samples are indicated inside each circle. *Pfhrp2/pfhrp3* status is indicated as: Positive/Positive (wild type), Negative/Negative (dual *pfhrp2/3* deletion*),* Positive/Negative *(pfhrp3* deletion*)* and Negative/Positive *(Pfhrp2* deletion)

In order to investigate the possible origin of *pfhrp2/3* gene deleted parasites, genetic relatedness analysis was conducted among 42 Ugandan parasite haplotypes as well as between Ugandan and Eritrean parasites. Among parasites from Uganda, there was a major cluster of parasites having deleted both *pfhrp2* and *pfhrp3* (H29, H30 and H31), and small clusters of *pfhrp2-/pfhrp3* + (H37 and H38), *pfhrp2* + */pfhrp3-* (H26 and H27) suggesting that these parasites are genetically closely related (Fig. 2a). There were several small clusters of closely related *pfhrp2* + */hrp3* + parasites. The biggest of clustering consisted of parasites with dual *pfhrp2/3* deletions indicating possible clonal expansion of these RDT undetectable parasites possibly selected for by RDT usage.

Haplotypes of Ugandan parasites (gene deleted and not deleted) did not cluster with parasite haplotypes from Eritrea (E1–E14), suggesting parasites with gene deletions in Uganda did not share common genetic origins with Eritrean parasites (different genetic lineages) and hence, were not likely to have resulted from spread from Eritrea. There is a trend indicating that parasites have formed small clusters within the Western and Eastern regions. However, there were two clusters that included parasites from both regions (Fig. 2b).

### Genetic diversity in the pfhrp2 gene

To investigate genetic diversity of the *pfhrp2* positive parasite population, Exon 2 of the *pfhrp2* gene was sequenced from 62 *pfhrp2* positive samples to reveal genetic diversity in the gene. Sequencing analysis revealed 60 unique sequence types in 62 samples sequenced, i.e. almost every sample had a different *pfhrp2* sequence, demonstrating a high level of genetic diversity in *pfhrp2* of the sequenced parasite population. Only two sequence types were shared between two different samples: the unique sequence types 39 (shared by D102 and D103) and 49 (shared by C041 and D165). Details are indicated in Additional file 2: Table S2.

### Sequence length of pfhrp2 exon2 which encodes the HRP2 protein

**Fig. 3:**
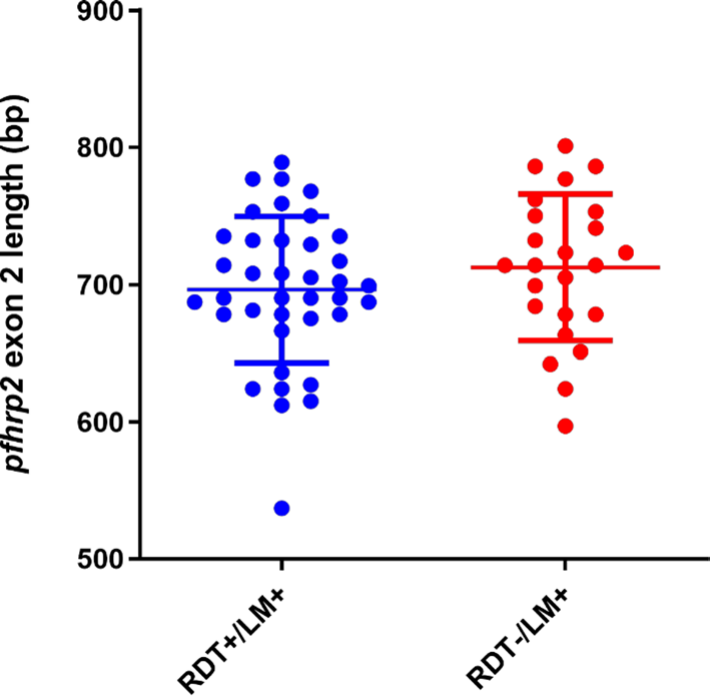
Sequence length of *pfhrp2* exon2. LM means light microscopy

Exon2 length of the 62 sequences ranged between 537 and 801 bp. Generally, sequence length in the two sample sub-sets was comparable between the RDT−/LM + and the RDT+/LM + samples (p = 0.28) (Fig. 3). Detailed data on *pfhrp2* exon length is included in Additional file 2: Table S2.

### Phylogeny and sequence clustering

**Fig. 4:**
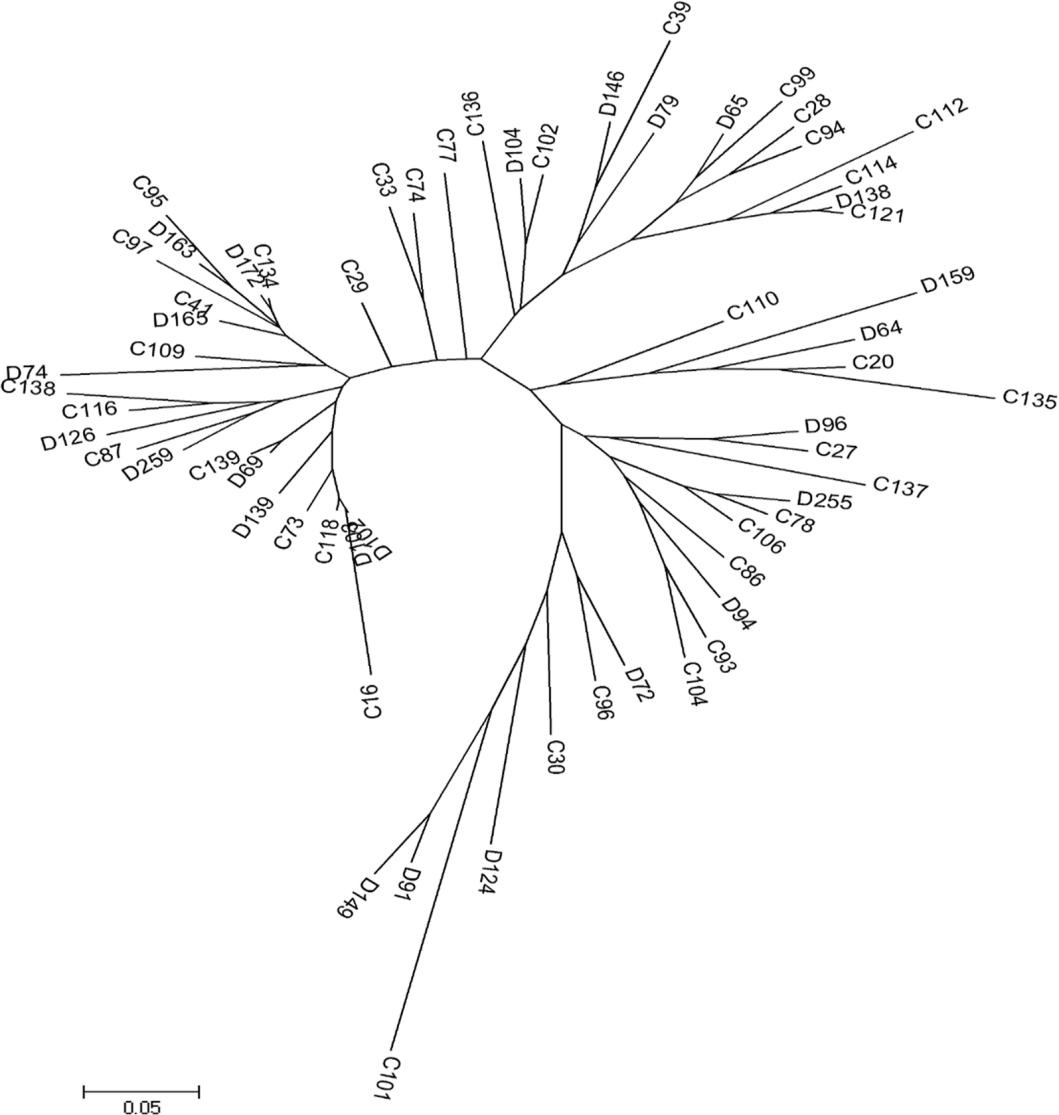
Phylogeny bootstrap consensus neighbour-join tree. D = discordant samples (RDT−/LM +), C = concordant samples (RDT+/LM +)

A phylogeny bootstrap consensus neighbor-join tree based on 62 *pfhrp2* exon2 sequences was constructed. No major clustering of parasites was observed in relation to RDT positivity, sequence length or sequence types. Interestingly, the three *pfhrp2* + */hrp3-* parasites (D126, D146 and C139) did not appear to be clustered as they are on 3 separate branches (Fig. 4). This suggests that parasites with *pfhrp3* deletions have emerged independently from different genetic backgrounds.

### Putative amino acid repeat types in HRP2

**Fig. 5:**
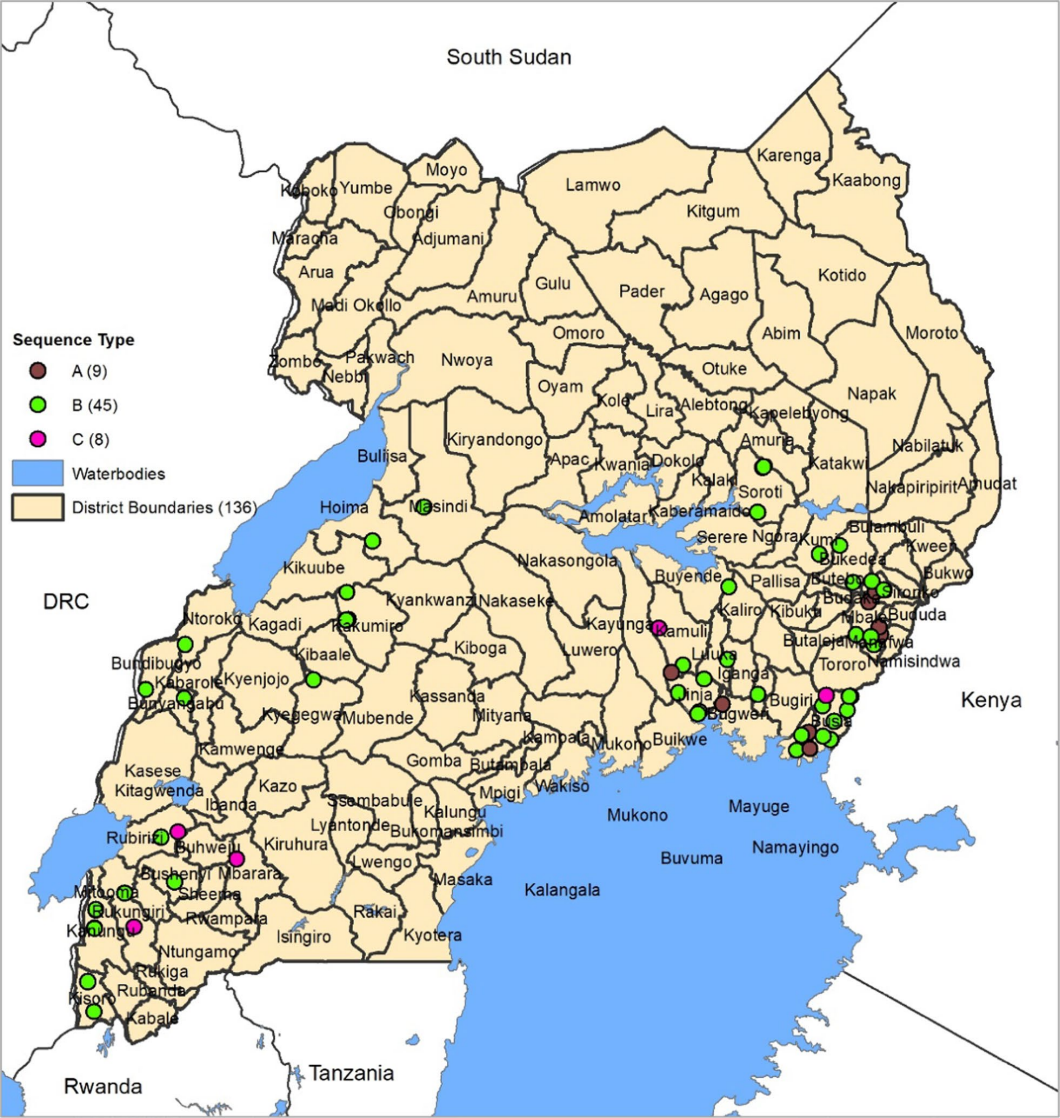
GIS mapping for distribution of sequences across sites. The different colored dots represent the different HRP2 sequence types

Among 62 sequences, 15 types of amino acid repeats were identified. Their numbers and ranges are shown in Table 4. There was no significant correlation between the number of any repeats and geographical origin or RDT positivity Table 4. The geographical distribution of the different HRP2 sequences is shown in Fig. 5.

**Table 4:** *pfhrp2* Amino Acid Repeat types, translated sequences and frequencies (n = 62)

PfHRP2		Total (N = 62)	Region		RDT and LM		p-value
			East	West	RDT−/LM +	RDT+/LM +	
Code	Repeat sequences		(N = 40)	(N = 22)	(N = 24)	(N = 38)	
1	AHHAHHVAD	2.7 ± 1.4	1–7	0–6	2.9 ± 1.5	2.6 ± 1.3	0.504
2	AHHAHHAAD	11.8 ± 2.2	10–17	8–13	12.1 ± 1.9	11.7 ± 2.4	0.427
3	AHHAHHAAY	1.2 ± 0.7	0–2	0–3	1.1 ± 0.7	1.3 ± 0.7	0.457
4	AHH	0.5 ± 0.8	0–3	0–1	0.3 ± 0.6	0.6 ± 0.9	0.146
5	AHHAHHASD	0.7 ± 0.5	0–2	0–1	0.8 ± 0.5	0.6 ± 0.5	0.221
6	AHHATD	3.0 ± 1.4	1–6	2–5	3.0 ± 1.3	3.0 ± 1.4	1
7	AHHAAD	6.5 ± 2.4	4–11	4–8	6.2 ± 2.1	6.8 ± 2.5	0.317
8	AHHAAY	1.1 ± 0.6	0–2	1–2	1.2 ± 0.8	1.1 ± 0.5	0.85
9	AAY	0.0 ± 0.0	0.0	0.0	0.0 ± 0.0	0.0 ± 0.0	NA
10	AHHAAAHHATD	1.6 ± 0.7	0–3	0–2	1.9 ± 0.7	1.5 ± 0.6	0.018
11	AHN	0.0 ± 0.0	0.0	0.0	0.0 ± 0.0	0.0 ± 0.0	NA
12	AHHAAAHHEAATH	0.0 ± 0.0	0	0	0.0 ± 0.0	0.0 ± 0.0	NA
13	AHHASD	0.0 ± 0.0	0	0	0.0 ± 0.0	0.0 ± 0.0	NA
14	AHHAHHATD	0.3 ± 0.4	0–1	0–1	0.4 ± 0.5	0.2 ± 0.4	0.097
*New		0.0 ± 0.3	0–2	0	0.0 ± 0.0	0.1 ± 0.3	0.324

*New = are sequences observed in Ugandan isolates that had not been reported before. East and West are the Eastern and Western regions of Uganda. Data was given by mean + -SD. P-value was calculated by Student’s t-test or Wilcoxon’s Rank Sum test as appropriate. LM = microscopy

### HRP2 Amino acid sequence grouping

The amino acid sequences were grouped following the previously published formula (Type 2 × type 7) [16]. Proportions of samples with Type A sequence (≥ 100), Type B (44–99) and Type C (≤ 43) were: 12.9% (8/62), 77.4% (48/62) and 9.7% (6/62), respectively. Similar to sequences reported from other countries [20] the majority of parasites in the Ugandan samples had a Type B *pfhrp2* sequence. Interesting to note is that 10% of HRP2 sequences are of type C that was initially reported to be associated with reduced performance of some HRP2-based RDTs [16] but later found to have no association when a larger sample size was analysed [20]. The proportion of Type C sequence accounted for 13% in the RDT−/LM + compared to 8% in RDT+/LM + samples (Table 5).

**Table 5:** Unique *pfhrp*2 sequences, sequence types and their proportions (n = 62)

Genomic DNA sub-sets	No. sequenced	Unique Sequence types identified		Sequence type A		Sequence type B		Sequence type C	
	Total	N	%	N	%	N	%	N	%
RDT+/LM +	38	38	100%	5	13%	30	79%	3	8%
RDT−/LM +	24	23	96%	3	13%	18	75%	3	13%
Overall	62	61	98%	8	13%	48	77%	6	10%

RDT+/LM + and RDT−/LM + are genomic DNA samples that were initially (HRP2 RDT negative and microscopy positive) and those that were both HRP2 RDT and microscopy positive respectively

## Discussion

Under the national malaria control and elimination strategic plan 2021- 2025, the Uganda National Malaria Programme aims to achieve malaria elimination in targeted districts by 2025. However incomplete surveillance data hampers efforts to assess parasite transmission dynamics and the impact of ongoing interventions. As *P. falciparum* population studies have revealed increasing genetic diversity and proportion of polyclonal infections correlating with increasing transmission intensity [29], molecular epidemiological surveillance of parasite diversity and relatedness has the potential to provide useful information for malaria transmission intensity, particularly in the elimination context where detecting ongoing local transmission and identifying the origin of infections may be relevant. The current study presents molecular characterization of the parasite population using microsatellite markers to determine genetic diversity and parasite relatedness with a special interest in the emergence of parasites with *pfhrp2/3* deletions, and use of genetic sequencing to investigate variability of the *pfhrp2* gene in *P. falciparum* populations from a wide geographical coverage and epidemiological settings in Uganda. The study is a follow up investigation that used genomic *P. falciparum* DNA from a previous survey that investigated and reported the presence of *pfhrp2/3* gene deletions in parasite isolates [23].

Microsatellites are abundant in *P. falciparum*; an average of one microsatellite locus is found every 2–3 kb of genome sequence [31, 32]. A set of 7 microsatellite markers that had been used to compare genetic relatedness between parasites with and without *pfhrp2/3* deletions in different areas of the world including South America and Eritrea were used, providing important information on whether parasites with gene deletions were of de novo emergence. For comparison purposes, exactly the same set of microsatellite markers were used in this study. The 3D7 parasite DNA was included in every microsatellite run as a common control for calibrating data. Using 7 Microsatellite loci scattered throughout the genome of *P. falciparum*, markedly high population-level parasite genetic diversity was found in the study samples that is consistent with malaria transmission intensities in the survey areas (Fig. 5).

### High proportion of polyclonal infections

In this study, most of the samples, 75.5% (95% CI 61.1–85.8), were of multiclonal (mixed genotype) *P. falciparum* infections. The proportion of samples with polyclonal infections observed in this study is higher than 60.2% (50/83) reported for Entebbe area in Uganda in the late 1990s also using microsatellite makers [29]. Using 12 microsatellite markers, Anderson et al. reported a strong positive correlation between proportion of polyclonal infections and transmission intensity: < 20% polyclonal infections were detected in low transmission settings of South America, intermediate proportions in Thailand, while > 45% polyclonal infections were detected in Africa and Papua New Guinea where malaria prevalence is high. Hence, the proportion of polyclonal infection observed in the current study indicates malaria transmission intensity remains high in Uganda. This conclusion is consistent with high malaria transmission levels reported in the study areas [5].

Previous studies in similar high transmission settings in Africa have reported similar high levels of multiclonal and genetically diverse *P. falciparum* infections [15, 33–37]. In contrast, other studies in Africa [38, 39], found a lower frequency of patients with multiclonal infections. This variation likely reflects heterogeneity in malaria transmission intensity resulted from geographical variability, seasonality and population variations. The frequency of multiclonal infections has also been reported to increase with age until late childhood before declining [35]. In this study, the isolates were collected from children between 2 and 10 years of age however no significant differences in multiclonal infections were observed between the < 5 and ≥ 5 years. This can be explained partly by the fewer samples tested in the < 5 age group (29%) vs 71% for > 5 age. Interestingly, multiclonal infections occurred more frequently in the Eastern compared to the Western region 73.7% (95% CI 48.8–89.1), P < 0.05). This observation is consistent with the relatively high level of malaria transmission in the Eastern region [5, 11, 14].

### High multiplicity of infection

MOI is defined as the number of genetically distinct parasite strains co-infecting a single host, which is an important indicator of malaria epidemiology [15, 40]. The overall MOI in the samples was 1.92 (95% CI 1.7–2.1). MOI did not differ between age groups < 5 and ≥ 5 groups (P = 0.60). This observation is in line with previous findings in Uganda that showed that parasitaemia is concentrated in children between 2 and 15 years of age which falls within the age groups (2–10 years) where the study samples were collected [41]. Although differences in MOIs were not significant between regions, the frequency of occurrence was higher in the Eastern compared to Western region (1.9 vs 1.8), consistent with the high transmission setting in the Eastern region [5, 11, 14]. Previous studies elsewhere in Africa have reported comparably high MOIs in parasite populations in similar transmission settings [21, 33, 40]. In Uganda, similarly high MOIs were reported in two localized studies at two hospitals in Kampala (MOI = 3.0) and Arua (MOI = 2.2) conducted 13 and 8 years ago, respectively [42, 43], when malaria prevalence in the country was much higher than recent years [5, 11, 12]. It is important to note however both studies used the MSP1 and MSP2 genes that are under immune selection pressure [15].

The number of co-infections within a host might be an important indicator of transmission intensity and annual incidence rates (API) and, therefore, a measure of impact of interventions [15]. In addition to determination of malaria transmission intensity, multiplicity of infections in *P. falciparum* are essential parasite indices that can impact on the effectiveness of malaria intervention due to their effect on treatment, diagnostics, surveillance and parasite transmission dynamics [15, 21]. There is indication that MOI can also help to monitor vaccine efficacy since vaccine-induced immune responses show strain-transcending specificity depending on the polymorphic alleles of the vaccine candidate antigens as reported previously by Zhong et al*.* [15]. The study reported greater RTS, S vaccine activity against malaria parasites with matched circumsporozoite protein allele than against mismatched parasite strains. A similar significance for studying MOIs is seen in therapeutic efficacy studies where detection of all clones is important, as only one of them might be drug resistant and will result in recrudescence. Non- detection of all clones at baseline might lead to false identification of these clones as new infections even when they existed at baseline, and thus an underestimate of treatment failure [15]. Several studies in Uganda have reported correlations between clinical symptoms and higher MOIs [42, 43]. Understanding MOIs in parasite populations in wider surveys could provide important information on transmission dynamics and inform current and future malaria control intervention efforts in Uganda.

### High HRP2 genetic diversity

Previous studies have reported the presence of *pfhrp2/3* gene deletions in Ugandan parasites, however genetic variation in the *pfhrp2* gene that may affect the functionality of HRP2-based RDTs has not been described on a wider scale [23, 44, 45]. Certain amino acid repeats within the *pfhrp2* gene have been shown to contain epitopes targeted by the monoclonal antibodies used to detect the HRP2 protein [16, 20, 46]. Variation in the pattern and sequence of histidine repeat tandems, number and frequency of repeat tandems and composition of amino acids within the HRP2 may affect the accuracy and functionality of HRP2-based RDTs [16, 20]. In the current study, the *pfhrp2* exon 2 was sequenced and examined to determine genetic variability of the *pfhrp2* gene in the samples. A high level of *pfhrp2* genetic diversity was found as revealed in 60 unique sequences from 62 samples sequenced 96.8% (60/62). This provides further evidence indicating high transmission intensities in the study areas.

While sequence length of exon2 of the *pfhrp2* that encodes the HRP2 protein antigen ranged between 537 and 801 bp in these samples, parasites with Type B sequences were the most common (48/62, 77.4%). Parasites with Type C sequences that usually contain fewer AHHAHHAAD and AHHAAD motifs which are possibly associated with reduced sensitivity of HRP2 RDTs [16, 18, 20, 47], were detected in low frequency (6/62, 10%) in the study samples. A slightly higher frequency of type C sequences was observed in RDT−/Microscopy + group compared to RDT+/microscopy + group (13% vs 8%) (Table 5). This may provide an additional explanation for the false negative RDT results not due to the quality of the RDTs or user related challenges reported by three previous studies in Uganda [23, 44, 45]. Further studies with larger sample size are required to validate this finding.

### Parasite genetic relatedness analysis

Despite recent advances in genome sequencing of malaria parasites, microsatellite markers remain an important source of data to understand the genetic diversity and relatedness of parasite populations and understanding of transmission dynamics [15, 21, 22, 32, 33, 48–51]. In this study, the genetic relatedness among monoclonal Ugandan parasites and between monoclonal Ugandan and Eritrean parasites was investigated using 7-microsatellite-marker haplotypes. The interest was to assess if there was any clustering among Ugandan parasites and in particular any clustering of Ugandan parasites with Eritrean isolates because of the high levels of *pfhrp2/3* gene deletions reported in Eritrea that led to the change of policy and replacement of HRP2 RDTs with non-HRP2-based RDTs [28] and because both parasite populations have been analysed by the same laboratory using the same set of microsatellite markers. It was assessed whether parasites with or without gene deletions in Uganda are genetically closely related to those reported in Eritrea. The analysis informs whether mutant parasites emerged locally or somehow spread from Eritrea. Analysis was performed using 42 unique haplotypes from 29 samples. A major cluster of parasites having deleted both *pfhrp2* and *pfhrp3* (H29, 30 and 31), and small clusters of *pfhrp2-/pfhrp3* + (H37 and H38), *pfhrp2* + */pfhrp3-* (H26 and H27) were observed suggesting that these parasites are genetically closely related. Clear clustering observed for *pfhrp2/pfhrp3* deleted parasites suggests clonal expansion of double deleted parasites due to selection. *Pfhrp2/3* gene deleted parasites in Uganda did not cluster with those from Eritrea (accessed using the same microsatellite markers), suggesting local emergence of gene deleted parasites. This observation suggests that the gene deleted parasites observed in Ugandan samples emerged independently by spontaneous genetic event rather than introduced through Eritrea. Future genetic studies on parasite populations in neighbouring countries may shed further light on the origin of gene deleted parasites in Uganda.

### Implications for detection of pfhrp2 deleted parasites

*Plasmodium falciparum* parasite with *pfhrp2* and *pfhrp3* gene deletions have been reported in Uganda [23, 44, 45]. It is critical to continuously assess the prevalence of gene deleted parasites to inform malaria control policies in the country. The high proportion of polyclonal infections and MOI > 1 detected in the parasite populations in study areas presents a major challenge to the gene deletion surveillance. Co-infection that involves a non-deleted strain can mask a gene deleted strain giving incorrect results when conventional PCR methods are used for detecting parasites with gene deletions [52, 53]. In the context of RDTs functionality, multiclonal infections involving wild type parasites and gene deleted parasites will give positive RDT results ensuring diagnosis and treatment of infected people, although this masks the presence of gene deleted parasites. This effect could occur even if an infection with a deleted parasite occurs subsequent to an infection with a wild type parasite since circulating HRP2 can persist for up to 3–4 months [53]. In addition, parasites having deleted *pfhrp2* only can be detected by some RDTs due to cross-reactivity of antibodies to HRP3 although at higher parasitaemias [16]. This can similarly mask the detection of *pfhrp2* deleted parasites but on the other hand help diagnosis of patients.

### General implication of findings

The higher proportion of MOIs and multiclonal infections observed in the study implies that these samples were mainly collected from high transmission settings suggesting persistent high transmission in these areas. This may call for strengthening control and intervention efforts. Importantly data obtained from this study can serve as baseline for assessing the impact of current and future control intervention measures. The presence of high proportion of multiclonal infections in these samples calls for improvement in the detection capacity of malaria diagnostic tools to overcome challenges associated with the detection all parasite clones as genotyping might falsely identify them as new infections if missed at baseline in therapeutic efficacy studies.

Previous studies in Uganda reported higher MOIs in Kampala and Arua in West Nile respectively [42, 43]. In both studies children infected with multiple strains were more likely to have treatment failure and severe disease suggesting that these multiclonal infections have a role in disease progression and severity. Similarly higher MOIs of 3.39 were seen in *P. falciparum* samples in Western Kenya [33]. The relatively high MOI detected in the study areas have likely contributed to the high malaria disease burden reported in the areas. Currently there is no point of care test that detects multiclonal and multiplicity of infection to assist case management.

The study showed high genetic diversity in the *pfhrp2* gene in Ugandan parasite population reflected in the presence of different types of repeats, as well as variations in sequence, repeat number, and arrangement of these repeats. The data provided further indication for high transmission intensity in the study areas. In addition, the observation of a slightly higher proportion of Type C HRP2 sequences that may be associated with reduced efficacy of HRP2 RDTS in the RDT−/microscopy + samples is of concern and may call for investigation of their occurrence in parasite isolates in other regions of Uganda.

It was observed in this study that parasites that harbored gene deletions formed clusters among Ugandan isolates suggesting close relatedness. However, these were genetically unrelated to the Eritrean parasites. This suggests that gene deleted parasites in Uganda have emerged independently by spontaneous genetic event and implies the potential for gene deletion to occur anywhere in Uganda under the selection of HRP2-based RDT use. This calls for periodic monitoring surveys.

### Limitations of the study

The study was limited by the fact that the *P. falciparum* isolates analysed were obtained from only two regions of Uganda and from two selected groups: RDT−/microscopy + and RDT+/microscopy + , which leaves the status genetic diversity, MOIs and parasite relatedness in other regions unknown. It is recommended that future surveillance programmes should consider a more representative sample covering all regions of Uganda. Due to resource constraint, the *pfhrp2* gene sequencing and microsatellite typing were performed on a limited number of genomic DNA samples. The study was unable to determine haplotypes for samples with polyclonal infections. Due to the high proportion of polyclonal infections and low parasite densities, haplotypes could only be determined for a relatively small number of samples.

## Conclusion

High level of genetic diversity was observed in *P. falciparum* parasites reflected in the frequency of multiclonal infections, multiplicity of infection and variability of the *pfhrp2* gene in these samples. These findings are consistent with the high malaria transmission intensity and endemicity in these settings despite the scaling up of malaria interventions. Genetic relatedness analysis suggested spontaneous de novo emergence and clonal expansion of *pfhrp2* deleted parasites that requires continuous close monitoring to inform malaria diagnosis and case management policies. Future molecular epidemiological surveys on parasite genomics that are more representative with wider coverage are recommended.

## Supplementary Information

**Additional file 1: Table S1.** Summary of samples successfully genotyped on 4–7 microsatellite markers (-s: single haplotype, -m2: 2 haplotypes, -m3: 3 haplotypes, -m4: 4 haplotypes)

**Additional file 2: Table S2.** Sequence length and unique sequence type of pfhrp2 exon 2.

## Data Availability

Data for this study is available and the row dataset has been uploaded as additional information with the manuscript.

## Abbreviations

HRP2: Histidine Rich Protein 2
*pfhrp2*: *Plasmodium falciparum* Histidine rich protein 2 gene
RDTs: Rapid diagnostic tests
MSP1: Merozoite surface antigen 1
MSP2: Merozoite surface antigen 2
PCR: Polymerase chain reaction
WHO: World Health Organization
LM: Light microscopy
MOI: Multiplicity of infection
DNA: Deoxyribonucleic acid
rRNA: Ribosomal ribonucleic acid
DBS: Dried blood spots
