# Supplementary material for: Genetic diversity and genetic relatedness in Plasmodium falciparum parasite population in individuals with uncomplicated malaria based on microsatellite typing in Eastern and Western regions of Uganda, 2019–2020
Source: Malar J. 2021 May 31;20:242. doi: 10.1186/s12936-021-03763-6 (PMC8165787; doi:10.1186/s12936-021-03763-6)
Supplement: Supplementary file 1 — Additional file 1: Table S1. Summary of samples successfully genotyped on 4–7 microsatellite markers (-s: single haplotype, -m2: 2 haplotypes, -m3: 3 haplotypes, -m4: 4 haplotypes) [file 12936_2021_3763_MOESM1_ESM.docx]

Supplementary Table 4. Summary of samples successfully genotyped on 4-7 microsatellite markers (-s: single haplotype, -m2: 2 haplotypes, -m3: 3 haplotypes, -m4: 4 haplotypes)

|  | No. of Ms successfully typed | No. of samples | Concordant (RDT+/Microscopy+) | Discordant (RDT-/Microscopy+) | No. of haplotypes |
| --- | --- | --- | --- | --- | --- |
| Haplotype determined and relatedness analysis | 7 | 7 | C8-m2, C15-m3, C19-s, C22-s, C27-s, | D255-s, D263-m2 | 11 |
|  | 6 | 8 | C4-m2, C18-m2, C20-s, C21-m2, C26-s, C30-m2, | D154-s, D262-s, | 12 |
|  | 5 | 14 | C5-s, C34-m2, C39-m2, C41-m2, C42-s, C119-m2, | D130-s, D169-m2, D176-m2, D196-m2, D201-s, D204-m2, D208-s, D252-m2, | 23 |
|  | total | 29 | 17 | 12 | 46 |
| Haplotype  Not determined | 7 | 7 | C1-m, C6-m, C11-m, C13-m, C43-m | D148-m, D159-m, |  |
|  | 6 | 5 | C9-m, C12-m, C14-m, C32-m, C33-m |  |  |
|  | 5 | 8 | C3-m, C29-m, C35-m, C38-m, C40-m, C90-m | D118-m, D170-m |  |
|  | total | 20 | 16 | 4 |  |
