# Supplementary material for: Genetic diversity and genetic relatedness in Plasmodium falciparum parasite population in individuals with uncomplicated malaria based on microsatellite typing in Eastern and Western regions of Uganda, 2019–2020
Source: Malar J. 2021 May 31;20:242. doi: 10.1186/s12936-021-03763-6 (PMC8165787; doi:10.1186/s12936-021-03763-6)
Supplement: Supplementary file 2 — Additional file 2: Table S2. Sequence length and unique sequence type of pfhrp2 exon 2. [file 12936_2021_3763_MOESM2_ESM.docx]

Supplemental Table 5. Sequence length and unique sequence type of pfhrp2 exon 2.

| **Sample ID** | **Exon 2 seq length ( bp)** | **Unique Seq type** | **Sample ID** | **Exon 2 seq length ( bp)** | **Unique Seq type** |
| --- | --- | --- | --- | --- | --- |
| C016 | 678 | 16 | C118 | 729 | 40 |
| C020 (ms-s) | 624 | 6 | C121 | 636 | 9 |
| C027 (ms-s) | 735 | 44 | C134 | 777 | 55 |
| C028 | 675 | 60 | C135 | 624 | 7 |
| C029 (ms-m) | 789 | 58 | C136 | 615 | 4 |
| C030 (ms-m2) | 627 | 8 | C137 | 735 | 45 |
| C033 (ms-m) | 690 | 23 | C138 | 612 | 3 |
| C039 (ms-m2) | 717 | 38 | C139 | 690 | 26 |
| C041 (ms-m2) | 753 | 49 | D069 | 651 | 11 |
| C073 | 705 | 31 | D072 | 699 | 27 |
| C074 | 666 | 13 | D074 | 786 | 56 |
| C077 | 699 | 28 | D079 | 801 | 59 |
| C078 | 732 | 42 | D091 | 714 | 34 |
| C086 | 678 | 17 | D094 | 714 | 35 |
| C087 | 678 | 18 | D096 | 786 | 57 |
| C093 | 777 | 54 | D102 | 723 | 39 |
| C094 | 750 | 48 | D103 | 723 | 39 |
| C095 | 708 | 32 | D104 | 684 | 20 |
| C096 | 681 | 19 | D124 | 678 | 14 |
| C097 | 732 | 43 | D126 | 705 | 30 |
| C099 | 714 | 37 | D138 | 678 | 15 |
| C101 | 537 | 1 | D139 | 777 | 53 |
| C102 | 708 | 33 | D146 | 663 | 12 |
| C104 | 759 | 50 | D149 | 714 | 36 |
| C106 | 687 | 21 | D159 (ms-m) | 624 | 5 |
| C109 | 702 | 29 | D163 | 741 | 46 |
| C110 | 690 | 24 | D165 | 753 | 49 |
| C112 | 768 | 52 | D172 | 750 | 47 |
| C114 | 687 | 22 | D255 (ms-s) | 762 | 51 |
| C116 | 690 | 25 | D259 | 642 | 10 |
